# Pathogenic mechanisms and regulatory factors involved in alcoholic liver disease

**DOI:** 10.1186/s12967-023-04166-8

**Published:** 2023-05-04

**Authors:** Chuyun Yan, Wanting Hu, Jinqi Tu, Jinyao Li, Qionglin Liang, Shuxin Han

**Affiliations:** 1 Department of Hepatobiliary Surgery, Anhui Province Key Laboratory of Hepatopancreatobiliary Surgery, The First Affiliated Hospital of USTC, Division of Life Sciences and Medicine, University of Science and Technology of China, Hefei, 230001 Anhui China; 2 MOE Key Laboratory of Bioorganic Phosphorus Chemistry & Chemical Biology, Key Lab of Microanalytical Methods & Instrumentation, Department of Chemistry, Center for Synthetic and Systems Biology, Tsinghua University, Beijing, 100084 China; 3 The First Affiliated Hospital of Wannan Medical College, Yijishan Hospital of Wannan Medical College of Wuhu, Wannan Medical College, Wuhu, 241000 Anhui China; 4 Xinjiang Key Laboratory of Biological Resources and Genetic Engineering, College of Life Science and Technology, Xinjiang University, Urumqi, 830046 China

**Keywords:** Alcoholic liver disease, Metabolic enzymes, Regulatory factors, Pathogenic mechanisms, Therapeutic implications

## Abstract

Alcoholism is a widespread and damaging behaviour of people throughout the world. Long-term alcohol consumption has resulted in alcoholic liver disease (ALD) being the leading cause of chronic liver disease. Many metabolic enzymes, including alcohol dehydrogenases such as ADH, CYP2E1, and CATacetaldehyde dehydrogenases ALDHsand nonoxidative metabolizing enzymes such as SULT, UGT, and FAEES, are involved in the metabolism of ethanol, the main component in alcoholic beverages. Ethanol consumption changes the functional or expression profiles of various regulatory factors, such as kinases, transcription factors, and microRNAs. Therefore, the underlying mechanisms of ALD are complex, involving inflammation, mitochondrial damage, endoplasmic reticulum stress, nitrification, and oxidative stress. Moreover, recent evidence has demonstrated that the gut-liver axis plays a critical role in ALD pathogenesis. For example, ethanol damages the intestinal barrier, resulting in the release of endotoxins and alterations in intestinal flora content and bile acid metabolism. However, ALD therapies show low effectiveness. Therefore, this review summarizes ethanol metabolism pathways and highly influential pathogenic mechanisms and regulatory factors involved in ALD pathology with the aim of new therapeutic insights.

## Background

Alcohol consumption is a widespread behaviour of people throughout the world. Approximately 2.4 billion people are classified as drinkers across the globe, with 960 million individuals classified as heavy drinkers [1]. Although moderate alcohol consumption may reduce the risk of cardiovascular diseases, long-term and excessive alcohol consumption can result in liver and nerve damage [2]. According to the World Health Organization 2018 Global Report on Alcohol and Health, excessive alcohol consumption kills approximately 3 million people annually across the globe, accounting for 5.3% of all deaths, and moreover, it is responsible for 47.9% of all cirrhosis-related deaths.

Ethanol is the main component of alcoholic beverages. Ethanol is mostly absorbed in the intestine, and after entering the bloodstream, it is oxidized and metabolized into acetaldehyde and acetic acid in the liver. This biochemical reaction results in the production of a large number of reactive oxygen species (ROS) and the destruction of the homeostatic environment in the liver. Over time, these changes can result in alcoholic liver disease (ALD).

ALD is characterized by liver injury, inflammation, fibrosis, cirrhosis, and/or cancer caused by long-term or large volumes of alcohol intake [3]. ALD is classified into several stages according to the pathological features of the liver. The first and most widespread stage, alcoholic fatty liver (AFL), is a result of increased lipid accumulation in the liver. The second stage, alcoholic hepatitis (AH), manifests as changes to hepatocytes that give them a balloon-like appearance, a large number of Mallory–Denk bodies, and liver infiltration by monocytes, neutrophils, and other inflammatory cells [4, 5]. ALD in the early stages can be alleviated by alcohol abstinence, but after continuous heavy alcohol consumption, ALD is irreversible. In the third stage, alcoholic liver fibrosis, collagen fibres extend into the lobules of the liver, and then, the final two stages of ALD, cirrhosis, and hepatocellular carcinoma, may develop.

Although 90% to 100% of long-term drinkers develop AFL, only 10% to 35% of these people develop alcoholic steatohepatitis (ASH). Furthermore, 8% to 20% of chronic heavy drinkers develop cirrhosis, with 2% of these patients developing hepatocellular carcinoma [3]. Although ethanol-induced liver injury has been of great concern in recent years, the underlying mechanisms of ethanol-induced liver injury are complex and involve multiple signaling pathways. Therefore, understanding these mechanisms is of great importance for the development of improved ALD treatments.

Alcohol-induced liver injury mainly involves structural damage to liver cells, lipid accumulation, and inflammation. Studies have demonstrated that transcription factors, kinases, and microRNAs (miRNAs) play critical regulatory roles in ALD. Herein, we summarize the enzymatic machinery involved in ethanol metabolism, followed by the molecular mechanisms thought to be critical to ALD and conclude with a summary of the therapies available for ALD.

## Ethanol metabolism

**Fig. 1:**
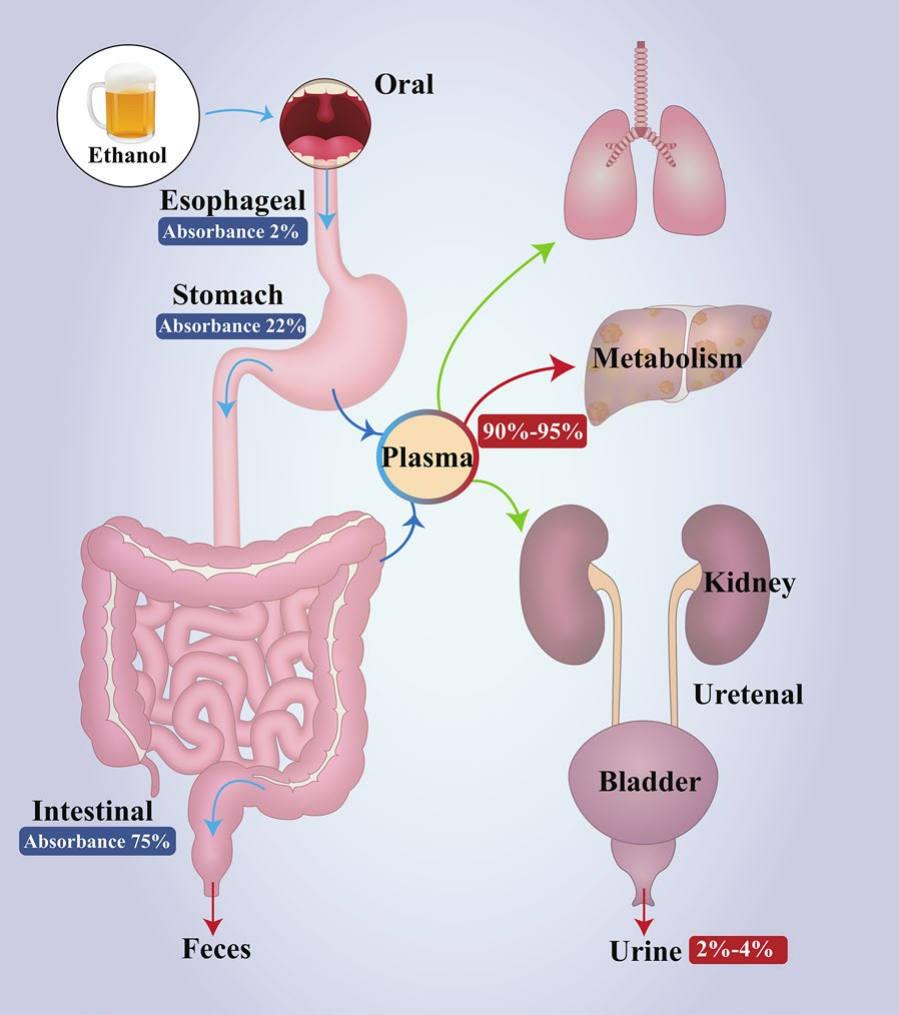
The absorption, transportation, and deposition of alcohol in the body. After oral ingestion, most alcohol is absorbed in the stomach (~ 22%) or the intestine (~ 75%), with only a small part deposited in faeces (1%) or absorbed in the oesophagus. The absorbed alcohol is transported mainly through the blood circulatory system to the liver where it is metabolized (~ 95%), with only a small portion excreted in urine (~ 2%-4%) or as an exhaled gas (~ 5%) in its original form

After being orally ingested, only a small amount of ethanol (2%) is absorbed in the mouth and oesophagus. Most of the ethanol is absorbed either in the stomach (22%) or the intestine (75%). Any ethanol not absorbed is excreted in faeces. Approximately 1% of the alcohol remaining in the body is excreted in its original form in the stool. After absorption, most of the imbibed ethanol enters the bloodstream and is metabolized by the liver. Only a small portion is removed by urine in its original form or exhaled via the lung in gaseous form (Fig. 1).

**Fig. 2:**
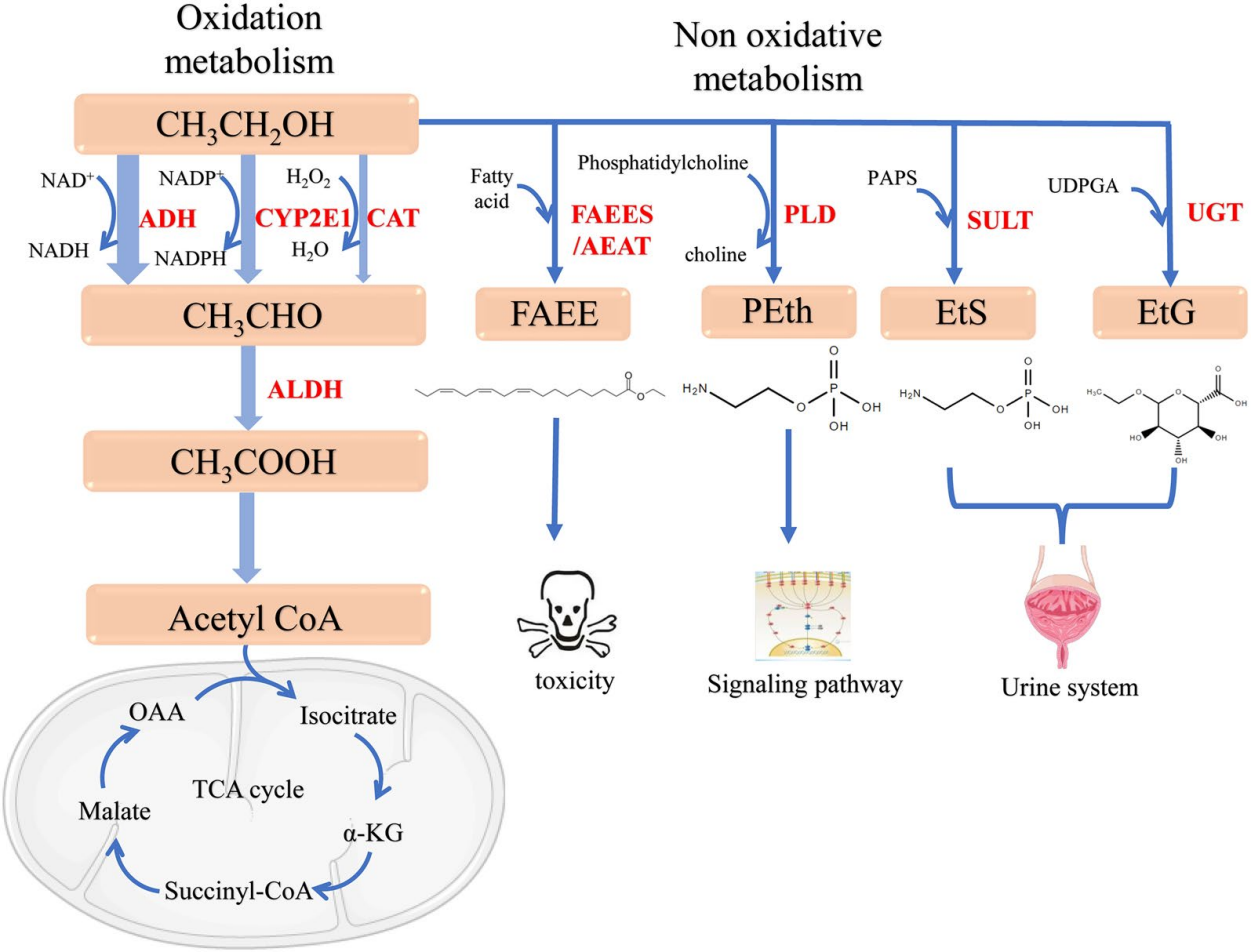
Ethanol metabolism pathways in the liver. In hepatocytes, three main oxidative pathways metabolize ethanol. Ethanol is metabolized to acetaldehyde by ADHs, CYP2E1, and catalase. Acetaldehyde is further metabolized into acetic acid by ALDHs. Acetic acid is then converted into acetyl-CoA by acetyl-CoA synthase, after which it enters the tricarboxylic acid cycle. Moreover, ethanol can be alternatively metabolized into the nonoxidative metabolites EtS, EtG, Peth, and FAEEs via the action of SULT, UGT, FAEES, AEAT, and PLD. EtS and EtG are water-soluble metabolites that are mainly excreted in the urine. *ADH* Alcohol dehydrogenases; *EtS* Ethyl sulfate; *EtG* Ethyl glucuronide; *PEth* Phosphatidylethanolfaees, fatty acid ethyl esters; *SULT* Sulfotransferase; *UGT* Glucuronosyltransferase; *FAEES* FAEE synthase; *AEAT* Acyl-CoA: ethanol O-acyltransferase; *PLD* Phospholipase ; *DCAT* Catalase; *ALDH* Aldehyde dehydrogenase; *OAA* Oxaloacetate

Ethanol, a two-carbon amphiphilic small-molecule compound, is oxidized and metabolized into an intermediate, acetaldehyde, by ethanol dehydrogenases, microsomal oxidases, and catalases in the liver (Fig. 2). In addition to being metabolized into acetaldehyde, ethanol metabolism leads to an increase in ROS levels and decreases in the nicotinamide adenosine dinucleotide (NAD)^+^/NADH ratio.

Under physiological conditions, most ethanol (80%–90%) is metabolized in the cytoplasm by alcohol dehydrogenases (ADHs) [5]. The main ADHs involved in ethanol metabolism are ADH1A, ADH1B, ADH1C, and ADH4 [6–8]. Haesba et al. reported that ADH5 may also be involved in ethanol metabolism [9]. The other 10 to 20% of ethanol is metabolized through hepatic microsomal ethanol oxidation. The main enzyme involved in this reaction is cytochrome P450 family 2 member E1 (CYP2E1). The expression level of CYP2E1 increases after ethanol consumption [10, 11].

Ethanol can be alternatively metabolized via nonoxidative pathways. Using 3'-phosphoadenosine 5'-phosphosulfate as a sulfonyl donor, less than 0.1% of ethanol is transformed by sulfotransferases (SULTs) into ethyl sulfate (EtS), which is readily excreted in urine [12]. SULT1A1, SULT1A3, SULT1B1, SULT1C2, and SULT2A1 in the liver show appreciable affinities for ethanol [13]. In one study, the expression levels of SULT1A1 and SULT2A1 in the liver and intestine of rats were increased after 2 weeks of ethanol intake [14]. From approximately 0.5% to 1.5% of ethanol is conjugated with uridine diphosphate glucuronic acid to produce ethyl glucuronide (EtG) under the catalytic action of uridine diphosphate (UDP)-glucuronosyltransferase (UGT) [15]. In addition, Hepatic UGT1A1, UGT1A3, UGT1A7, UGT1A6, UGT2B4, UGT2B7, UGT2B15, and UGT2B17 can utilize ethanol as a substrate [13]. For example, one study demonstrated that UGT1A1 expression was significantly upregulated in chronic ethanol-imbibing rats, while UGT1A5 and UGT2B1 expression was only slightly increased [16]. Another study revealed that the expression of UGT1A1, UGT1A5, UGT2B1, UGT2B3, and other UGTs is highly upregulated [17–19]. Ethanol can be esterified with fatty acids (FAs) to produce fatty acid ethyl esters (FAEEs) [20]. Esterification is primarily mediated by fatty acyl synthases, which are expressed mainly in the liver and pancreas [21]. In the presence of ethanol, the choline in phosphatidylcholine (PC) can be replaced with ethanol to produce phosphatidylethanol (PEth), which is a highly sensitive blood-tested indicator of ethanol intake [18, 19].

After conversion from ethanol, acetaldehyde is oxidized to acetic acid by aldehyde dehydrogenases (ALDHs). Acetaldehyde metabolism is the key rate-limiting step in the alcohol oxidative metabolism pathway and is mediated by ALDH1A1, ALDH1B1, and ALDH2 in humans [22]. However, recent studies have shown that ALDH2 metabolizes acetaldehyde to produce acetic acid not only in the liver but also in cerebellar astrocytes. In the cerebellum, an ALDH2-mediated enzymatic reaction increases the level of gamma-aminobutyric acid, leading to mobility and balance disorders [23]. Finally, acetic acid is conjugated with coenzyme A (CoA) through the action of acetyl-CoA synthase to form acetyl-CoA, which can enter the tricarboxylic acid cycle or participate in other reactions, such as gluconeogenesis, ketone formation, amino acid formation, and FA synthesis (Fig. 2).

## Mechanisms of ethanol-induced liver diseases

**Fig. 3:**
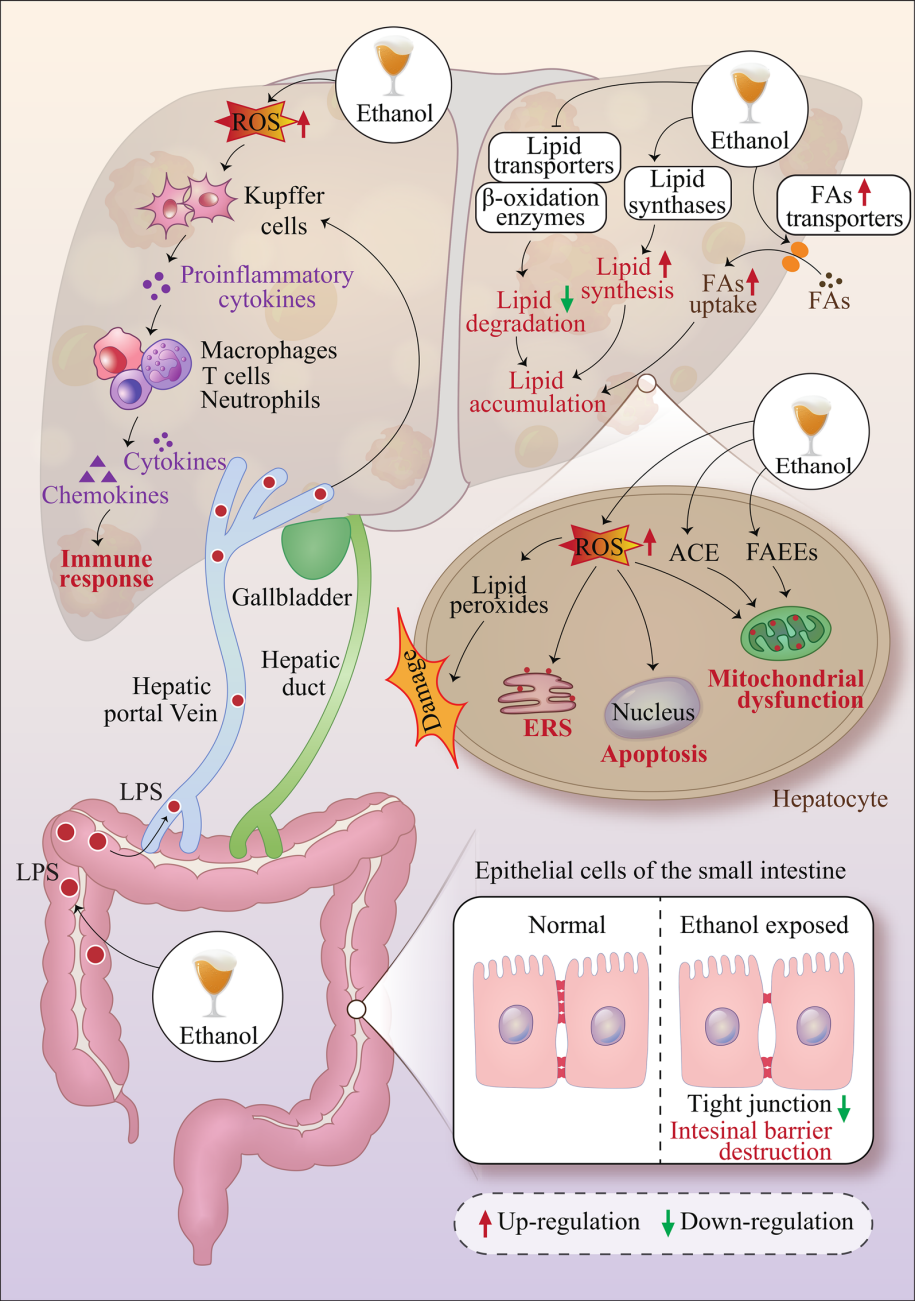
Mechanisms of ethanol-induced liver injury. Ethanol can upregulate lipid synthase and FA transporter activity to increase lipid synthesis and downregulate lipid transporters and β-oxidases to reduce lipid consumption, which eventually leads to lipid accumulation. ROS produced by ethanol oxidative metabolism triggers the release of proinflammatory cytokines and activates a variety of immune cells to release chemokines and cytokines, leading to immuno-inflammatory responses. Ethanol also inhibits tight junctions to disrupt the intestinal barrier, inducing intestinal LPS translocation to further stimulate the inflammatory response in the liver. In hepatocytes, ROS and lipid peroxidation products generated during ethanol metabolism activate oxidative stress and ER stress reactions in multiple organ systems. ACE and FAEEs, metabolites of ethanol, also induce mitochondrial dysfunction. *ROS*, reactive oxidative species; *LPS* lipopolysaccharide; *FAs* fatty acids; *ER stress* Endoplasmic reticulum stress; *ACE* acetaldehyde; *FAEEs* Fatty acid ethyl esters

Several mechanisms are possibly associated with ethanol-induced liver diseases. For example, acute ethanol consumption can result in oxidative stress, nitrification stress, endoplasmic reticulum (ER) stress, inflammation, and apoptosis. Chronic ethanol consumption can result in dysregulated lipid metabolism, lipid accumulation, and AFL and alter the gut-liver axis by damaging the tightly connective structures of the intestinal epithelium (Fig. 3).

### Mitochondrial damage

Mitochondria harbour many redox systems, and this redox enzyme system is an early target of ethanol. Clinical data suggest that approximately 25% of patients with ALD present with mitochondrial melanosis due to mitochondrial hyperplasia [24].

Ethanol exposure can reduce mitochondrial volume and the F0 adenosine triphosphate (ATP) synthase levels and inhibit protein synthesis. Ethanol metabolism can also disrupt the phospholipid bilayer membrane structure of mitochondria, resulting in the release of a large number of oxygen free radicals, lipid peroxidation, unsaturated FA destruction, and the production of malondialdehyde (MDA) and 4-hydroxynonenal (4-HNE) [25–28]. Although the destruction of unsaturated FAs damages the integrity of the mitochondrial membrane, the formation of macromolecular complexes due to the condensation of MDA with amino acids or phospholipids markedly decreases the fluidity of the mitochondrial membrane [29].

The oxidative metabolite of ethanol, acetaldehyde, is highly toxic and can readily undergo crosslinking with DNA or protein macromolecules in mitochondria. After accumulation in cells due to heavy ethanol consumption, acetaldehyde leads to mitochondrial dysfunction by crosslinking to mitochondrial DNA and mitochondrial proteins and inhibiting the electron transport chain [30].

Notably, some nonoxidative metabolites produced by ethanol metabolism can cause damage to the mitochondrial membrane. For instance, FAEEs can destabilize the mitochondrial membrane, disrupt the flow of electrons in the respiratory chain and inhibit oxidative phosphorylation [31]. Moreover, Aydin et al. showed that FAEEs induced the apoptosis of HepG2 cells reduced the number of cells in the G2/M and S phases, and interfered with the cell cycle [32]. Interestingly, FAEEs can be bound, synthesized, and hydrolyzed by human platelets, resulting in bleeding or clotting [33].

### Oxidative stress

Ethanol oxidation leads to an increase in free radical production in the liver. Uncontrolled ROS are critical to hepatocyte swelling and apoptosis [34]. ROS can interact with DNA, proteins, and lipids, disrupting numerous metabolic and homeostatic cellular functions. The metabolism of ethanol mediated by CYP2E1 in liver cells is closely related to excessive ROS production and NADPH oxidase subtype NADPH oxidase 4 (NOX4) overexpression [35, 36].

Excessive ROS can alter the function of glutathione (GSH) transporter channels, leading to progressive GSH deficiency in mitochondria and inactivation of oxidative phosphorylase, resulting in an irregular mitochondrial shape, decreases in mitochondrial protein synthesis, and altered ATP production in mitochondria [37–39]. In addition, ROS depleted cellular antioxidant stores [40]. Additionally, ROS promotes the formation of lipid peroxidation products, such as MDA and 4-HNE. MDA and 4-HNE can react with DNA bases to form DNA adducts that form outwards-oriented DNA loops. Specifically, MDA reacts with deoxyguanosine residues, and 4-HNE reacts with deoxyadenosine and deoxycytidine [41, 42]. These interactions promote apoptosis and necrotic cell death, contributing to the progression of ethanol-induced liver diseases.

Moreover, ethanol alters signalling cascades, such as the Wnt/β-catenin signalling pathway [43]. Long-term ethanol consumption increases the nuclear expression of β-catenin and the cytoplasmic expression of p-GSK-3β, Wnt2, and Wnt7a [44]. Interestingly, pretreatment with maltomanopeptide, a PI3K/Akt pathway inhibitor, significantly reduced the levels of liver triglycerides (TGs) [45]. Additionally, clinical data suggest that low-density lipoprotein receptor-associated protein 6 is a potential upstream mechanism underlying ethanol-induced activation of the Wnt/β-catenin signalling pathway [46].

Toll-like receptor 4 (TLR4) signalling cascades are altered by ethanol, leading to the activation of myeloid differentiation primary response 88 (MyD88) and an inflammatory response [47]. The association of MyD88 with interleukin (IL)-1 receptor-associated kinases 1–4 leads to the activation of the tumour necrosis factor receptor-associated factor 6/transforming growth factor β-activated kinase 1 complex, which activates mitogen-activated protein kinases (MAPKs), c-Jun N-terminal kinase (JNK), P38, and extracellular signal-regulated protein kinase (ERK), resulting in inflammation and apoptosis [48, 49]. In addition, ethanol consumption can result in nuclear factor-kappaB (NF-κB) translocation to the nucleus via the phosphorylation of inhibitory κB (IκB) and to the release of inflammatory cytokines.

Acetaldehyde can induce ROS production and lead to ALD [50]. Specifically, the binding of acetaldehyde to GSH inhibits the scavenging function of hydrogen peroxide, thereby exacerbating oxidative stress and lipid peroxidation [51]. Additionally, Oh et al. showed that acetaldehyde induced an increase in p38 and JNK phosphorylation in PC12 cells, while penicillin reversed these reactions and prevented oxidative stress caused by acetaldehyde [52]. Farfan Labonne et al. also demonstrated that acetaldehyde reduced the ATP content, respiratory control, superoxide dismutase (SOD) activity, and glutathione/oxidized GSH ratio [53].

In addition to aggravating oxidative damage in the liver, FAEE accumulation can lead to pancreatitis [54]. Furthermore, the concentration of FAEEs (20–40 μM) in the blood has been shown to induce oxidative stress and reduce mitochondrial function in intestinal epithelial cells [55].

### Nitrification stress

Nitrification stress refers to the biochemical action of reactive nitrogen species, where nitric oxide (NO) is attached to a tyrosine residue, resulting in the generation of intracellular proteins carrying 3-nitrotyrosine (3-NT) residues [56]. 3-NT can in turn function as a new antigen and stimulate the production of autoantibodies against the proteins that harbour it, thus mediating certain autoimmune diseases. 3-NT may also exert a variety of additional toxic effects on cells, leading to cell damage and apoptosis [57, 58].

The main enzymes related to nitrification stress are two subtypes of NO synthase (NOS): endothelial NOS (eNOS) and inducible NOS (iNOS). Under physiological conditions, eNOS is abundant in the liver, whereas the iNOS level is barely detectable. Yuan et al. demonstrated that chronic ethanol intake increases the NO level, particularly through the expression of iNOS, but decreases eNOS activity in the liver [59]. These ethanol-induced changes in NO and nitrification stress levels are mediated through the hypoxia-inducible factor 1 (HIF1) [60]. Activated HIF1 induces iNOS production of NO, which is then decomposed into peroxynitrite. The increased nitrification of tyrosine kinases can lead to alterations in the MAPK and JNK signalling pathways, resulting in the apoptosis of liver cells.

Argininosuccinate synthetase (ASS) is a rate-limiting enzyme in the L-citrulline/NO pathway. The intake of a large amount of ethanol induces the expression of ASS, which enzymatically activates the nitrification stress response and apoptosis in liver cells. The specific loss of ASS can cause resistance to nitrification stress caused by acute ethanol exposure [61, 62]. Ethanol also initiates NF-κB-mediated transactivation of iNOS expression in hepatic Kupffer cells. Furthermore, Nanji et al. reported that blocking lipopolysaccharide (LPS)-induced activation of NF-κB effectively alleviated ethanol-induced liver injury [63].

In addition to oxidative stress, ROS plays important role in nitrification stress. CYP2E1 inhibition or iNOS knockdown in mice or prevention of oxidative/nitrifying stress can reduce the activation of JNK and P38. CYP2E1 induced by pyrazole or ethanol increased the production of superoxide radicals, while ethanol-increased LPS/tumour necrosis factor-α (TNF-α) activated iNOS and NO production [64].

### ER stress

ER stress reduces the concentration and aggregation of unfolded proteins. Sustained ER stress may lead to cell and tissue damage. Three canonical ER stress signalling pathways are the activating transcription factor 6 (ATF6), inositol-requiring enzyme 1 (IRE1), and protein kinase R-like ER kinase (PERK) pathways [65]. PERK, IRE1, and ATF6 may be phosphorylated and activated after dissociation from the immunoglobulin proteins to which they are bound to trigger ER stress responses [66, 67].

Szczesna et al. found that CYP2E1 activity induced by high-dose ethanol promoted both the unfolded protein response and ER overload response [68]. Nuclear translocation of ATF6 was observed in ethanol-treated hepatocytes, and lipid overload and ethanol treatment were found to be conducive to ER stress [69]. The ER stress marker tribbles homologue 3 has also been found to be increased after ethanol exposure [70]. Many studies have identified an association between ER stress and microapoptosis [71, 72]. Ethanol can trigger the ER stress response, which is followed by the stimulation of apoptosis-related gene expression, cell proliferation, and apoptosis imbalance. Under severe ER stress conditions, the PERK–eukaryotic initiation factor 2α (eIF2α)–ATF4–C/EBP-homologous protein (CHOP) signalling pathway is activated. Silencing the CHOP gene reduced hepatocyte apoptosis and hepatic fibrosis in patients with ALD [73, 74]. Moreover, some studies have suggested that homocysteine is the major cause of misfolded protein aggregation in the ER. For example, Outinen et al. found that homocysteine treatment altered the expression of ER stress-related genes such as CHOP and sterol-regulatory element-binding protein (SREBP) in endothelial cells [75]. Specifically, increases in homocysteine were correlated with ALD [76]. In ethanol-treated rats, the expression of ER stress-related proteins and homocysteine levels were increased [77]. In addition, the levels of the oxidative stress-related proteins CYP2E1, SOD2, glutathione-S-transferase (GST) inhibitor-2, and NAD(P)H:menadione oxidoreductase were elevated. Interestingly, the anti-apoptotic protein Bcl-2 level was decreased. In the liver, homocysteine can be methylated to methionine by methionine synthase and betaine-homocysteine methyltransferase. As reported by Cheng et al., methionine synthase levels are reduced in animals after a two- to four-week period of ethanol consumption, increasing the liver homocysteine content [78].

Similar to ROS and nitrification stress, acetaldehyde contributes to ER stress. Lluis et al. found that acetaldehyde impaired mGSH transport and sensitized HepG2 cells to TNF-α action through an ER stress-mediated increase in cholesterol [79]. Acetaldehyde also stimulated an increase in mitochondrial cholesterol content, which resulted in a time- and dose-dependent reduction in the mGSH level [80].

In addition, ER stress induced by ethanol plays a significant role in the development of tumours [81]. The PERK-eIF2α-ATF4 pathway activates the expression of angiogenic genes and vascular endothelial growth factors. Prolonged expression and activation of ATF6 increased the Rheb homologue protein abundance in the brain, specifically in the mechanistic target of the rapamycin (mTOR) signalling pathway, and prolonged tumour cell survival. The inositol-requiring enzyme 1 alpha/X-box-binding protein 1 pathway was shown to interact with members of the anti-apoptotic Bcl-2 family and sigma-1 receptors. These findings suggested that ER stress induced by ethanol potently stimulated tumour growth and angiogenesis. The same study also characterized tumour necrosis factor-α (TNF-α)-induced ER stress as a homocysteine-independent pathway. Notably, the administration of betaine was found to effectively reduce ethanol-induced homocysteine and ER stress in the liver, thereby alleviating ALD [82].

### The ethanol-induced “second-hit” theory of ALD

#### Lipid accumulation

AFL is the first and most widespread stage in the development of ALD. Numerous studies have revealed that ethanol significantly induces fat accumulation and steatosis in the liver. Fat is composed mainly of glycerol and FAs. Under normal physiological conditions, the FA pool level is low and stable, but ethanol can lead to an imbalance in the FA pool. The two main sources of FAs are exogenous FAs, which are utilized by the body after digestion, and endogenously synthesized FAs, in which pyruvate from glycolysis is consumed for de novo FA synthesis. Both in vivo and in vitro experiments have shown that ethanol upregulates FA uptake and plasma membrane expression in the liver [83, 84]. The uptake of FAs is mediated by FA transporters. CD36/FA transposase (FAT), FA transport protein (FATP), and FA-binding protein are three major types of FA transporters that play important roles in ethanol-mediated lipid synthesis and transportation in the liver [85]. In vivo, ethanol exposure increases the mRNA and protein levels of CD36/FAT and FATP5. Ethanol exposure also increases the protein levels of microsomal triglyceride transfer protein (MTP) but decreases the levels of apolipoprotein (Apo) B100 mRNA and protein [86]. In addition to altering the levels of FA transporters, ethanol exposure can result in the accumulation of NADPH, thereby promoting FA de novo synthesis. Increases in the NADPH level result in changes in AMP-activated protein kinase (AMPK) signalling and inhibition of ATP consumption, which is a prerequisite for the synthesis of FAs. Therefore, the inhibitory effect of ethanol on AMPK phosphorylation can effectively stimulate the synthesis of FAs [87].

In addition to increasing the FA pool, ethanol-induced lipid accumulation by inactivating enzymes involved in β-oxidation and inhibiting FA consumption. Acyl-CoA oxidase-1 (ACOX-1) is an important rate-limiting enzyme in the β-oxidation pathway, and activation of ACOX-1 effectively attenuated ethanol-induced hepatocyte steatosis [88]. The inhibitory effect of ethanol on peroxisome proliferator-activated receptor-alpha (PPARα) activity also suppressed β-oxidation. Some studies have revealed that ethanol-induced malnutrition reduced the transcription of carnitine palmitoyl transferase-1 (CPT-1), which facilitates the transport of FAs to mitochondria during β-oxidation. This effect may have been due to the impaired activity of AMPK, which mediated the release of acetyl-CoA carboxylase (ACC) inhibition, thus attenuating the activity of CPT-1. MTP and CPT-1 can be activated by activated AMPK and PPARα signalling to prevent ethanol-induced liver injury [89]. Taken together, studies indicate that ethanol inhibits β-oxidation directly or indirectly, ultimately leading to lipid accumulation.

A growing consensus among scientists suggests that polyunsaturated FAs (PUFAs) play important roles in the modification of membrane structures in the alcoholism context. PUFAs, especially omega-3, are beneficial to health. PUFAs are important stabilizing components of phospholipids in cell membranes. In contrast with saturated FAs, unsaturated FAs are less stable and can readily induce lipid peroxidation. Ethanol consumption changes the amounts of PUFAs in the liver [90, 91]. Stearoyl-CoA desaturase 1 (SCD1) is a delta-9 FA desaturase that catalyses the synthesis of monounsaturated FAs (MUFAs), major components of TGs, and ethanol treatment increases the expression of SCD1. Wang et al. demonstrated that inhibition of SCD1 activity attenuated alcoholic liver injury in female mice [92, 93]. SCD1-knockout (KO) mice are resistant to AFL development, and hepatic TG accumulation, activation of de novo adipogenesis, and increases in inflammatory marker levels are inhibited [94]. A diet enriched with saturated FAs has been shown to reduce endotoxaemia, lipid peroxidation, and TNF-α and cyclooxygenase-2 (COX2) levels, effectively preventing ethanol-induced liver damage, including fibrosis [95]. These findings indicate that ethanol exposure leads to the loss of membrane PUFAs and functional damage to cells [96]. Notably, the effect of ethanol on FA synthesis appears to be evolutionarily conserved, as the same reduction process has been identified in *Escherichia coli* [97]. The activity of lipoprotein lipase (LPL) increases after ethanol consumption and is upregulated in a tissue- and diet-dependent manner. This increased LPL activity, in turn, increases the high-density lipoprotein (HDL) cholesterol level in the liver [98]. Clinical trials performed to evaluate the effects of 4 weeks of ethanol consumption showed that chylomicron TG and HDL cholesterol levels were significantly increased and that the low-density lipoprotein (LDL) cholesterol level was reduced in blood, whereas, the LPL level in adipose tissue was elevated [99]. Another study found that the levels of circulating LDL and HDL cholesterol were reduced [100]. Very-low-density lipoprotein (VLDL) secretion has been found to be altered [101]. In summary, the downregulation of lipid transporter action leads to lipid accumulation in hepatocytes.

Acetaldehyde plays a role in the development of a fatty liver. For example, Jokelainen et al. identified an alteration in lipid metabolism that resulted in fat accumulation in the livers of animals treated with acetaldehyde [102]. Moreover, Lemasters et al. showed that ethanol and acetaldehyde reduced the oxidation rate of FAs by mediating voltage-dependent anionic channel closure in the mitochondrial outer membrane [103]. The reduced FA oxidation rate is considered to be a potent pathogenic cause of fat accumulation. Finally, a clinical analysis of ADH1B and ALDH2 genetic polymorphisms showed that diminished ethanol and acetaldehyde metabolism accelerated the development of AFL in heavy drinkers [104].

#### Immune stress response

Ethanol exposure induces the production of many inflammatory cytokines and chemokines [88]. Clinical studies have shown that interleukin-1β, C–C motif chemokine ligand 2, monocyte chemoattractant protein 1, and macrophage migration inhibitory factor levels were significantly increased in the liver and blood of ALD patients [105, 106]. ROS produced from ethanol metabolism in hepatic Kupffer cells induced the production of large amounts of the proinflammatory cytokines IL-1β and TNF-α, which can activate natural killer T cells, macrophages, neutrophils, and a series of other downstream immune responses [107]. The inflammatory cascade function in tandem with ROS-induced activation of the NF-κB-inducing kinase/IκB kinase/IκB-α pathway [108]. TNF-α, IL-1β, iNOS, COX2, chemokines, adhesion molecules, and a series of immunoinflammatory factors produced through the effects of ethanol lead to immunoinflammatory and liver injury responses [109, 110].

In the early stages of ALD, suppression of tumorigenicity 2 receptor (ST2) inhibits the activation of hepatic macrophages to prevent injury. However, in late-stage ALD, a high rate of cell death is associated with IL-33/ST2 signalling [111, 112]. Toll-like receptors (TLRs) are also involved in the ethanol-induced inflammatory response [113]. TLR2 and TLR9 cause ethanol-mediated liver injury by inducing chemokine (C-X-C motif) ligand 1 expression and neutrophil infiltration. TLR3 in endosomes and exosomes is activated by myofibroblast transdifferentiated RNA to mediate the inflammatory response in ALD patients [114]. Activated M1 macrophages produce a large number of cytokines, such as IL-1β, TNFα, IL-12, IL-18, and IL-23, and induce antigen-specific helper T cell 1 (Th1) and Th17 cell inflammation [115]. Neutrophil infiltration is a marker of severe ASH.

The number of neutrophils in the liver and circulatory system is significantly elevated in mice fed ethanol for a long time [116, 117]. In contrast to macrophages and neutrophils, which initially appear to be beneficial, T-cell infiltration is thought to be harmful. An increase in the CD4 and CD8 T-cell subpopulations has been observed in the livers of ALD patients. Alcoholic cirrhosis promotes the progression of ALD via enhanced production of interferon-gamma, and a greater proinflammatory Th1 cell response has been identified found in the AH patients than in the alcoholic cirrhosis context [115].

### Ethanol causes liver damage mediated through the gut-liver axis

The liver and digestive tract are closely associated and their interactions form the gut-liver axis. The translocation of bacterial or microbial products from the intestine to the liver is a key inflammatory factor that induces the transition from alcoholic steatosis to ASH [118, 119]. Bile salts and antimicrobial molecules such as immunoglobulin A and angiopoietin in the liver are transported through the biliary tract, where they enter the intestinal lumen and modulate microbial growth and homeostasis. The intestine, in turn, reabsorbs secondary bile acids formed from primary bile acids by gut bacteria and transports these bile acids and other gut metabolites back to the liver via the portal vein.

Metabolites and inflammatory mediators generated from the interaction between the liver and intestine are released into the circulatory blood system. Therefore, more intensive attention has been recently focused on investigating the important roles played by the intestinal microbiota in ALD.

The intestinal flora is composed of various microorganisms, including aerobic bacteria such as *Bifidobacterium*, *Bacteroides*, and *Streptococcus* as well as anaerobic bacteria such as *Enterobacter*, *Enterococcus*, and *Lactobacillus*. Therefore, the vast microbial community forms a complex intestinal microorganism environment. Direct contact between ethanol and the gut after drinking may alter the composition of the microbiome in the gut. Furthermore, when the intestinal barrier is disrupted, microorganisms and their metabolites in the intestine may migrate to the liver via blood in the portal vein and destroy the health of the liver [120].

#### The damaging effects of ethanol on the gut barrier

Ethanol exposure results in the loss of epithelial cells at the tip of intestinal villi [121]. The gut barrier mainly regulates the water balance and nutrient absorption rate and selectively protects intestinal microorganisms [122]. The gut comprises four barrier layers: intestinal luminal alkaline phosphatase released by intestinal epidermal cells, surface mucus, the upper cortex, and the immune defence system [123]. A tight junction (TJ) complex is a major component of the intestinal barrier and consists of adherent junction proteins (e.g., E-cadherin and β-catenin), TJ proteins (e.g., zonula occludens 1 [ZO-1] and occludin), and cytoskeletal proteins. The complex protects against the entry of microorganisms into the intestinal lumen and prevents pathogens from entering the circulatory system [55].

After long-term ethanol intake, the intestinal epithelial cell structure in rats is markedly changed, exhibiting mitochondrial and endoplasmic reticulum dilation [124]. An in vitro study in which Caco2 cells were exposed to ethanol showed that adherent junctions and TJs were damaged by oxidative stress and nitrification stress [125]. The oxidative stress initiated by ethanol led to the oxidation of cytoskeletal microtubules and TJ decomposition. Moreover, ethanol caused the upregulation of NF-κB activity, destabilization of F-actin, downregulated expression of the miR-212-targeted gene ZO-1, and TJ destruction [126, 127]. Furthermore, nitrification stress-induced iNOS mediated the expression of the mesenchymal cell-related transcription factor Snail [128]. Interestingly, acetaldehyde was an inducer of more severe TJ damage than its precursor, ethanol. Acetaldehyde induced the redistribution of occludin and ZO-1 and the rearrangement of E-cadherin and β-catenin [129, 130]. Although ethanol destroys the intestinal barrier by damaging the tightly connective structures in the intestine, the intestine harbours an innate defence mechanism against ethanol-induced injury. Specifically, as reported by Park et al., ethanol stimulated the proliferation of intestinal epithelial cells through Wnt signalling-stimulated intestinal epithelial regeneration [131], which may be a defensive mechanism in the gut that prevents ethanol-induced damage.

#### Ethanol stimulates endotoxaemia and immunoinflammatory responses

LPS, an endotoxin derived from gram-negative bacteria located in the digestive tract, is a central pathogenic mediator of ASH. After travelling through the portal blood to the liver, LPS triggers a proinflammatory response by binding to TLRs. TLRs have been shown to play important roles in ALD progression. TLRs are infection sensors that induce immune responses after they bind to microbial cell walls. LPS activates TLR4/CD14 and a series of downstream targets, such as senescence-associated receptor PK, JNK, ERK1/2, and p38, through the myeloid differentiation factor 88–dependent/independent MAPK signalling pathway. LPS-initiated activation further induces the expression of activating protein-1 and NF-κB [132].

This process results in inflammasome activation with the production of inflammatory factors such as TNF-α, IL-1β, IL-6, and NLRP3the activation of an ER stress responseand hepatocyte death [133]. TLR3 in endosomes and exosomes can be activated by myofibroblast RNAs to trigger immune inflammatory responses in ALD patients [114]. Additionally, the TLR4-induced M1 polarization of Kupffer cells exacerbates the release of proinflammatory cytokines, and chemokines, and ROS promotes hepatic steatosis and apoptosis, together accelerating the development of AH and liver fibrosis [134].

Yan et al. identified a disruption in the homeostatic balance of bacterial populations in the gastrointestinal (GI) tract of mice fed ethanol for 3 weeks [135]. Although the abundance of the gram-positive bacteria *Firmicutes* decreased, the abundance of gram-negative species such as *Bacteroidetes* and *Verrucomicrobia* increased. Another study found that ethanol consumption caused gram-negative bacteria to produce a large amount of endotoxin, thereby increasing intestinal permeability and the translocation of endotoxin into the blood circulatory system [136]. This outcome was confirmed in another study, in which ethanol-fed mice showed higher endotoxin contents in the portal vein, hepatic vein, and pulmonary aorta blood, suggesting that ethanol may exert a significant challenge to the liver, reducing its ability to remove endotoxins [137]. Clinical data have also demonstrated this outcome, revealing that the level of endotoxin in the bloodstream is fivefold higher in ALD patients than in a control cohort [138]. Therefore, the multiple damaging effects of ethanol on the GI tract lead to intestinal endotoxaemia (Fig. 3).

#### Ethanol alters bile acid metabolism and intestinal flora contents

Bile acids are the main metabolites of cholesterol. They are synthesized in the liver and are critical for lipid-soluble nutrient digestion, absorption, and transportation. Studies have shown that ethanol increases bile acid synthesis in human, mouse, and rat livers [139, 140]. Farnesoid X receptor (FXR) has been considered a critical regulator of ALD, as indicated by FXR-deficient mice susceptible to developing ASH. Furthermore, FXR agonists effectively reduce the abnormally high level of bile acids in ALD patients [141].

The physical interaction between ethanol and intestinal microbes may be a key reason for the ethanol-driven alteration in the intestinal flora contents. The physiological diversity of bacteria was lost in ethanol-fed mice [142]. Moreover, in a study of intestinal bacteria in patients with alcoholic cirrhosis, ALD-related dysbiosis mainly manifested as *Lactobacillus* deficiency and *Candida* overgrowth [143]. Another study showed that the levels of *Enterococcus faecalis* were 2700-fold higher in faecal samples taken from ALD patients, and the parameters were associated with the severity and mortality of patients suffering from liver diseases such as AH [144]. Ethanol can also induce *Enterococcus faecalis* migration to the liver, where these bacteria secrete a large amount of cytolysin, damaging liver cells [145, 146]. Nonetheless, intestinal microorganism species are quite different between rodents and humans. Therefore, the experimental results of rodent ALD models need to be interpreted with caution.

## Important regulatory factors of ALD

**Fig. 4:**
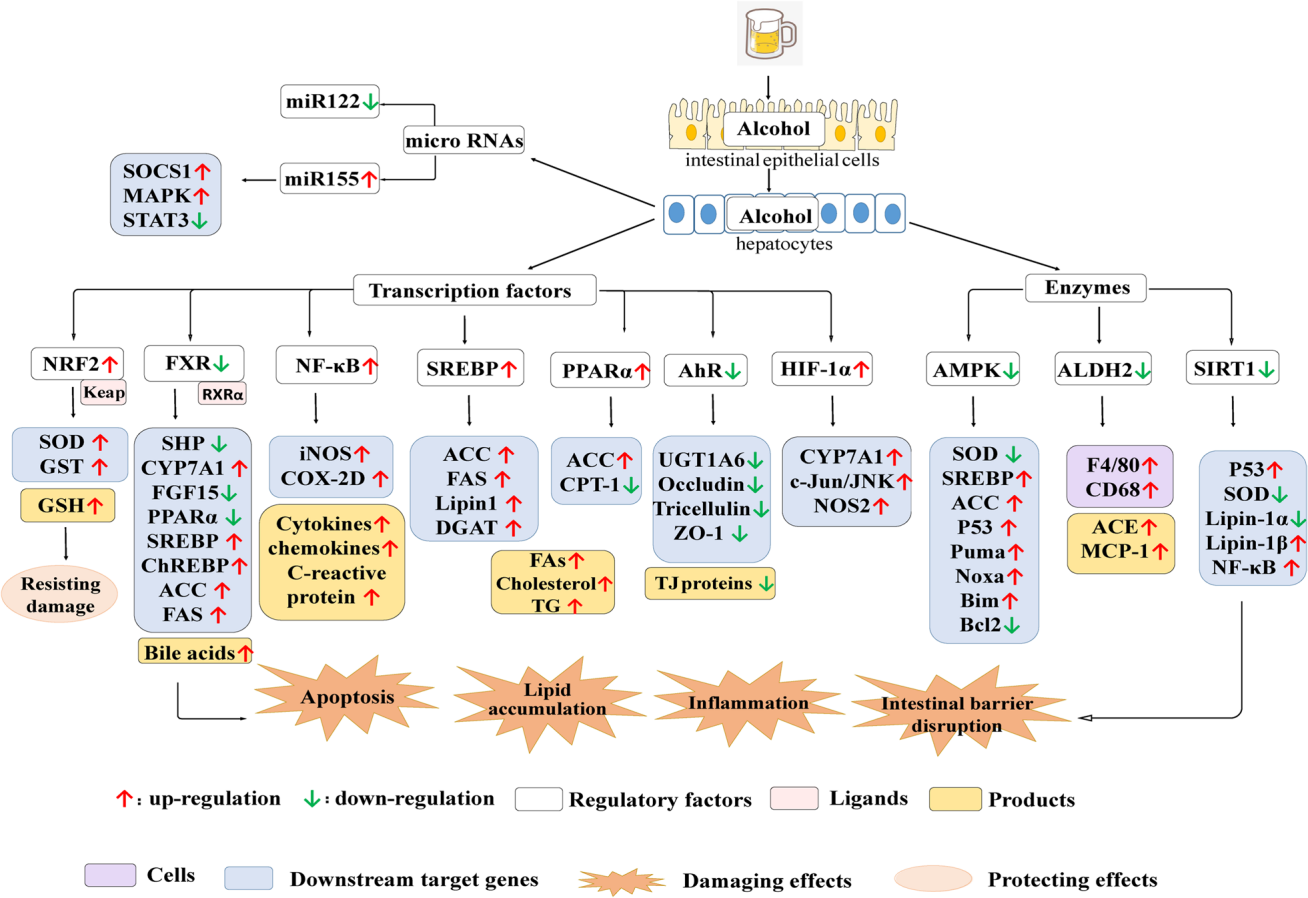
Regulatory factors in ALD. Ethanol is absorbed by villous epithelial cells in the intestine and is mostly transported to the liver for metabolism. Ethanol downregulates AMPK, SIRT1, miR-122, and FXR activity, increases the expression of lipid synthesis genes, and inhibits PPARα-mediated β-oxidation of fatty acids (FAs), leading to ethanol-induced fat accumulation in hepatocytes. The upregulation of miR-155 but suppression of AMPK initiated by ethanol activates apoptosis pathways in the liver. Increased bile acid synthesis due to ethanol-induced inhibition of FXR also accelerates hepatic apoptosis. The upregulation of NF-κB by ethanol stimulates the release of inflammatory factors and chemokines, leading to alcoholic hepatitis (AH). The body develops defences against the toxic effects of ethanol. For example, NRF2 is induced in hepatocytes by ethanol and promotes adaptive resistance to ethanol-initiated oxidative stress. *SIRT1* sirtuin 1miR-122, microRNA-122FXRPPARαmiR-155, microRNA-155AMPK, AMP-activated protein kinaseNF-κB, nuclear factor-kappaBNRF2, nuclear factor E2-related factor 2

Increasing evidence indicates that certain regulatory factors may play central roles in ALD. Numerous studies have indicated that many important regulatory factors, including transcription factors, biochemical enzymes, and miRNAs in the liver, are altered in the ALD context. These factors play important roles in the pathogenesis of ALD by regulating antioxidants, lipid metabolism, inflammation, and apoptosis (Fig. 4).

### Enzymes

#### ALDH2

ALDH2 is a key enzyme crucial to acetaldehyde catabolism in the liver. The daily production of acetaldehyde is reduced by approximately twofold in ALDH2-deficient patients [147]. Approximately 8% of the worldwide population and from approximately 30% to 40% of the East Asian population carry an inactive ALDH2 gene [148]. Ethanol has been reported to stimulate the expression of ALDH2, and moderate ethanol consumption likely exerts a protective effect on the cardiovascular system mainly through ethanol-activated ALDH2, sirtuin1 (SIRT1)/p53 pathway, and eNOS, which protect endothelial cells against ethanol-induced damage [149]. ALDH2 may be regulated at the transcriptional level and can be inactivated by SIRT3-mediated deacetylation [150, 151]. ALDH2 deficient activity causes extensive accumulation of reactive acetaldehyde and lipid peroxides, which interact with cellular macromolecules, including DNA/RNA, lipids, and proteins. These interactions lead to DNA mutations, ER stress, mitochondrial dysfunction, steatosis, and cell death.

Many in vivo experiments have confirmed that ALDH2 efficiently increases the survival rate of ethanol-fed mice by inhibiting ethanol-induced inflammatory responses and steatosis [152]. Mice carrying the dysfunctional ALDH2 mutant (E487K) were highly sensitive to acute and chronic exposure to ethanol-induced toxicity, and the mutant mouse strains exhibited a much lower survival rate under the same feeding conditions as the wild-type mice [153]. ACE accumulation and a significant reduction in white blood cells in the blood, hepatocyte apoptosis, and ataxia were observed in the ALDH2 (E487K)-expressing mice after moderate ethanol consumption. The increase in the level of MDA-acetaldehyde in ALDH2-deficient mice mainly led to Kupffer cell activation in the macrophage population, which caused an increase in the production of the cytokines monocyte chemoattractant protein-1 (MCP-1), IL-6, and TNF-α [154]. Zhong. et al. found that by feeding wild-type C57 mice ethanol for 8 weeks, ALDH2 lost its protective effect, and acetaldehyde accumulated in the experimental animals [155]. In the same study, further administration of the ALDH2 agonist Alda-1 reduced the acetaldehyde level and promoted FA oxidation in the liver, effectively ameliorating steatosis in the mice.

### SIRT1

SIRT1 is an NAD^+^-dependent deacetylase in mammalian cells. The expression of the enzyme is activated when the ratio of NAD^+^/NADH is high. SIRT1 is an important regulatory enzyme that modifies the protein activities of downstream factors via deacetylation to control the expression of target genes involved in the regulation of inflammation, lipid metabolism, and oxidative stress [156].

Early studies showed that the expression of SIRT1 was reduced in mice exposed to ethanol. Furthermore, this decline was more significant in ageing mice [157, 158]. SIRT1 regulates different lipin-1 subtypes, which constitute a group of magnesium-dependent phosphatidate phosphatase enzymes in mammals. Lipin-1β is a cytoplasmic enzyme in the liver that converts phosphatidyl acid to diacylglycerol, promoting the synthesis of TGs and phospholipids in the ER. Nucleoplasmic lipin-1α mediates the oxidation of FAs via the regulation of peroxisome proliferator-activated receptor-γ coactivator-1α and PPARα [159]. SIRT1 represses lipin-1β through the SIRT1–serine/arginine-rich splicing factor 10-lipin-1β/α axis and activates lipin-1α to inhibit lipid synthesis and promote FA oxidation [160]. SIRT1 also regulates AMPK levels [161].

SIRT1 can stimulate the expression of antioxidant enzymes such as SOD through the deacetylating activation of the β-catenin and forkhead box O proteins [162]. In turn, β-catenin mediates the expression of genes such as cyclin D1, which is a critical regulator of cell proliferation and survival and myelocytomatosis. Liu et al. reported that β-catenin-deficient mice were more susceptible to ethanol-induced liver damage [163].

SIRT1 has also been associated with the p53 signalling pathway. In particular, SIRT1 activates p53-mediated apoptosis via its deacetylation modification function [164]. SIRT1 deacetylates NF-κB and suppresses NF-κB transcriptional activity to maintain the low expression of inflammatory genes [165].

### AMPK

AMP-dependent protein kinase is a key molecule in the regulation of bioenergy metabolism. AMPK is activated via the phosphorylation of AMP, liver kinase B 1 (LKB1), and calmodulin-dependent protein kinase-β. Chronic ethanol exposure mainly inhibits AMPK by blocking the phosphorylation of the upstream kinases PKC and LKB1 [166].

In vivo studies have shown that ethanol inhibits AMPK phosphorylation [167, 168]. In particular, AMPK directly phosphorylates SREBP-1c, resulting in the inhibition of its transcriptional activity [169]. Ethanol can inhibit AMPK phosphorylation, thereby promoting lipin1, ACC, SREBP-1c, and carbohydrate response element-binding protein (ChREBP) [170–172]. Ethanol upregulates the expression of SREBP-1 and lipin-1 to promote the expression of lipid synthesis genes by inhibiting AMPK, resulting in an increase in newly generated lipids [170, 171]. Furthermore, the inhibitory effect of ethanol on AMPK and the upregulation of its downstream molecules SREBP, ACC, and lipin-1 mediate abnormal adiponectin signal transduction and inhibit the lipid-lowering effect of AMPK [173].

In addition to being an energy adapter, AMPK exhibits other physiological functions in cells. Notably, AMPK can be activated by ROS, which seems to contradict the inhibitory effect of ethanol on AMPK activity [174]. However, researchers have found that ROS activation of AMPK may be related to the balance between the rate of ROS formation (inducing AMPK phosphorylation and activity) and ethanol-induced inhibition of AMPK functions. Colombo et al. reported that AMPK induced the expression of antioxidant proteins such as manganese-SOD, catalase, glutamylcysteine synthetase, and thioredoxin [175], which may represent a self-regulating mechanism to eliminate excess ROS.

AMPK may increase either cell survival or apoptosis depending on the signalling pathways activated by AMPK [176]. Shin et al. demonstrated that AMPK promoted cell survival by inhibiting glycogen synthase kinase-3β or activating forkhead box O 3A (FoxO3A) [177]. In vitr*o* experiments showed that globular adiponectin mediated FoxO3A translocation into the nucleus via the AMPK-FoxO3A axis and activated autophagy, thereby attenuating the ethanol-induced apoptosis of HepG2 cells [178]. However, prolonged activation of AMPK can trigger apoptosis. AMPK counteracted cell proliferation but induces cell cycle arrest and apoptosis by suppressing mTOR/Bcl-2 and activating p53/PUMA/NOXA and Bim [179–182].

Cilostazol, a phosphodiesterase 3 inhibitor, protects liver cells against ethanol-induced apoptosis by activating the AMPK pathway [183]. In addition, previous studies have shown that high levels of ethanol consumption reduced the autophagy rate through the action of AMPK by promoting ROS production or by inhibiting mTOR/rapamycin pathway activation [184, 185]. Calcitriol, a vitamin D analogue, alleviated ethanol-induced hepatotoxicity through AMPK/mTOR-mediated autophagy [186]. In summary, long-term ethanol abuse inhibits autophagy, leading to severe liver damage [187].

### Transcription factors

#### SREBP

SREBP functions as an intracellular lipid sensor and regulates a wide range of FAs and TG synthases, such as ACC, fatty acid synthase (FAS), and cholesterol CoA dehydrogenase [188, 189]. Among the three subtypes of SREBPs, SREBP-2 is involved mainly in cholesterol synthesis, whereas SREBP-1a and SREBP-1c are involved in FA biosynthesis [190, 191]. Knocking out SREBP-1 results in a decrease in FA synthesis but higher SREBP-1-mediated cholesterol production [192].

In vivo experiments demonstrate that acute and chronic ethanol intake upregulates the expression of SREBP-1 in mice. Ethanol-induced ER stress increased SREBP-1 activation to increase lipid synthesis [193–195]. In parallel, in vitro experiments have shown that cultured hepatocytes exposed to ethanol exhibit an increased level of active nuclear SREBP-1 [17]. Specifically, Chen et al. proved that long-term excessive ethanol intake stimulates the expression of SREBP through the mTOR/S6 kinase 1/SREBP-1c pathway [196].

Several SREBP pathways are induced by ethanol, such as the inflammatory response and ER stress pathways. Endo et al. demonstrated that oxidative stress-triggered TNF-α increased the expression of SREBP-1c mRNA in mouse liver and the nuclear fraction of active SREBP-1 in the human liver [197]. The expression of SREBP was also upregulated by ethanol-mediated ER stress. The ER stress response results in the dissociation of ATF6 from immunoglobulin protein. Free ATF6 is then translocated to the Golgi body, where it is cleaved by site-1 protease and site-2 protease. Active ATF6 then enters the nucleus [198, 199].

In addition, ethanol exposure induces heat shock protein 90 to promote the expression of nuclear SREBP, SREBP-1, ACC-1, and stearoyl-CoA desaturase-1. Therefore, drug inhibition of heat shock protein 90 can alleviate AFL [200, 201]. ACC, FAS, and cholesterol CoA dehydrogenase are the target genes downstream of SREBP [188, 189]. Diacylglycerol transferase (DGAT) 1 and DGAT2 activated the biosynthesis of TGs and their expression was upregulated in ethanol-treated mice [92]. Ethanol increased lipin1 expression through AMPK-SREBP-1 signalling [160]. Through these mechanisms, high expression of SREBP induced by ethanol stimulates FA synthesis and lipid accumulation in the liver, leading to the formation of AFL.

#### PPARα

As first reported by Green et al., PPARα is a nuclear hormone receptor/nuclear transcription factor activated by ligands that regulate FA oxidation and transport [202]. When activated, PPARα forms a heterodimer with retinol X receptor (RXR), and this dimer binds to the peroxisome proliferative response element, which is included in a set of genes involved in lipid metabolism [203, 204]. PPARα exhibits a protective effect on ethanol-induced liver damage. PPARα-KO mice were more sensitive to ethanol-induced liver injury than wild-type mice [205]. Another in vivo study revealed that activated PPARα efficiently ameliorated fatty liver in rats after intragastric administration of ethanol [206].

Several reasons likely explain the ethanol-induced downregulation of PPARα activity. For example, PPAR/RXR activity is negatively regulated by the ethanol-inhibited AMPK/SIRT-1/peroxisome proliferator-activated receptor-γ coactivator 1α (PGC-1α) signalling axis [207]. In addition, ethanol-induced oxidative stress may lead to the overproduction of TNF-α and the downregulation of PPARα expression [208, 209]. Ethanol may also inhibit PPARα via the action of ROS derived from CYP2E1-mediated oxidation [210].

The inhibition of PPARα led to the upregulation of ACC expression and the inhibition of malonyl-CoA decarboxylase expression, which was followed by increased malonyl-CoA production, inhibited CPT-1 activity, and reduced FA oxidation. These results indicate that PPAR is a potential therapeutic target for ALD.

#### NRF2

Nuclear factor E2-related factor 2 (NRF2) is a transcription factor expressed via a set of cell defence-associated target genes, including genes that encode various antioxidant enzymes, drug-metabolizing enzymes, and molecular chaperones DNA repair enzymes such as peroxidase, SOD, GST, and GSH [211, 212]. Because its expression is tightly regulated under antioxidant-induced stress conditions, NRF2 may be closely related to ALD, as indicated by liver-specific deficiency of NRF2 accelerating ethanol-induced mortality and liver injury [213].

Ethanol is thought to induce the activation of NRF2 because of the increase in oxidative metabolism caused by ethanol-initiated stress. Under physiological conditions, NRF2 is sequestered by the inhibitory factor Kelch-like ECH-associated protein 1 (KEAP1) [212]. After the induction of oxidative stress, NRF2 dissociates from KEAP1 in response to electrophile signals or phosphorylation by several kinases, such as PKC, ERK, and phosphoinositide 3-kinase regulatory subunit 3 [214]. Activated NRF2 then enters the nucleus, interacting with musculoaponeurotic fibrosarcoma protein to form a heterodimer, which then binds to antioxidant response elements, modulating the transcription of target genes [215, 216].

In addition to regulating the expression of phase I drug metabolism enzymes, NRF2 is involved in regulating a variety of phase II detoxification enzymes, such as UGT and SULT, in the liver [217]. Although a role for NRF2 in the direct regulation of UGT and SULT during ethanol metabolism has not been described in relevant studies, some drug toxicological studies have proven that drug administration normalizes UGT activity and restores UGT2B expression through the action of the NRF2 signalling pathway [218].

In addition to controlling downstream enzyme expression, NRF2 plays an important role in supporting the integrity of mitochondria, particularly under stress conditions [219]. NRF2-KO mice presented with more severe damage to mitochondrial structure and function [220]. Wang et al. also reported that NRF2 boosted the level of VLDL receptors in the liver [221]. This finding may explain TG accumulation in liver cells.

In addition, gut NRF2 plays an important role in ALD. Ethanol consumption leads to the destruction of the intestinal barrier and migration of LPS and pathogenic microorganisms into the liver [222]. The bacterial metabolite urolithin A/UAS03 significantly upregulated NRF2 and TJ proteins such as claudin 4, NAD(P)H quinone dehydrogenase 1, occludin, ZO-1, and TJ protein 3. Importantly, however, urolithin/UAS03 failed to upregulate TJ proteins in NRF2-KO mice. These results support the idea that NRF2 is important for maintaining the expression of intestinal epithelial TJ proteins as well as the intestinal barrier. Furthermore, ethanol exposure can change the composition of the intestinal flora, as the *Lactobacillus* level has been shown to be reduced in the intestine of ALD patients. Therefore, using tandem mass spectrometry, Bejan et al. analysed blood from the portal vein in mice carrying *Lactobacillus rhamnosus GG* [223]. Notably, the authors found that *Lactobacillus intestinalis* produced 5-methoxy-indoleacetic acid, which stimulated NRF2 expression and led to resistance to the acute toxicity induced by ethanol. In summary, NRF2 possibly regulates the enterohepatic axis in the attenuation of ALD during treatment.

#### NF-κB

NF-κB is a master transcription factor that regulates inflammatory responses by promoting the production of inflammatory factors and hepatocyte apoptosis. NF-κB is bound and its action is inhibited by the inhibitory factor IκBα under physiological conditions. Ethanol can induce the expression and activation of NF-κB, often depending on ROS induction [224, 225]. Ethanol-induced oxidative stress and ER stress lead to NF-κB activation and the expression of downstream inflammatory target genes. Additionally, ethanol-mediated activation of Kupffer cells results in the production of the cytokine TNF-α and exacerbates ROS production. In response to external stimuli (e.g., ROS), TLR/myeloid differentiation factor 88, IL-1 receptor (IL-1R)/IL-1R-associated kinase, or TNF receptor/TNF receptor-associated factor 2 is activated, IκBα is ubiquitinated and dissociates from NF-κB, and then, free NF-κB can enter the cell nucleus to exert transcriptional regulation.

Studies have shown that NF-κB plays an important role in regulating the expression of downstream inflammatory genes [226]. NF-κB can induce the expression of cytokines IL-4, IL-6, IL-1β, and TNF-αthe chemokines macrophage inflammatory protein-1 and IL-8the enzymes iNOS and COX-2Dvascular cell adhesion molecules 1 and 2and the proinflammatory compound C-reactive protein [227–232]. Chemical inhibition of NF-κB activity reduces the levels of IL-6, TNF-α, alanine aminotransferase (ALT), aspartate aminotransferase (AST), iNOS, and COX-2 but increases the levels of SOD and GSH in the liver or blood of ALD individuals [233–235]. As a consequence, inflammatory factors mediate the apoptosis of liver cells.

#### HIF1-α

HIF1-α, a key factor in the response to hypoxic stress, is a transcription factor evolutionarily conserved in mammals. HIF1 is a heterodimeric transcription factor consisting of an oxygen-sensitive α subunit and a constitutively expressed β subunit. The heterodimer transcription factor, first discovered by Semenza while studying erythropoietin binds to hypoxia response elements in target genes [236]. After stimulation by hypoxia, α subunits enter the nucleus where they dimerize with HIF1-β to transactivate their target genes [237].

Bernstein et al. and Israel et al. found that oxygen consumption by the liver was increased in mice subjected to long-term chronic ethanol intake [238]. The expression or DNA-binding activity of hepatic HIF1-α was enhanced in ethanol-fed mice [239]. Moreover, HIF1-α liver-specific KO mice were more resistant to ethanol-induced liver damage [240].

Activation of HIF1-α is related to oxidative stress induced by CYP2E1-driven ethanol metabolism [241]. Superoxide anion-driven oxidative stress promotes HIF1-α activation and the expression of iNOS. Under physiological conditions, nitric oxide synthase 2 (NOS2) remains at a low level, but ethanol metabolism-induced oxidative stress stimulates the gene expression of NOS2 [242]. NOS2 enzymatically converts L-arginine to L-citrulline, and this conversion is accompanied by the production of a large amount of NO, which then forms the reactive nitrogen molecule nitric dioxide and peroxynitrite under oxidative stress. Peroxynitrite mediates the nitrification of tyrosine residues in proteins to form 3-NT and activates the phosphorylation of JNK to accelerate cell apoptosis [243, 244]. HIF1-α overexpression increases the activity of the cholesterol 7α-hydroxylase (CYP7A1) promoter [245], which may be downstream mechanisms of ethanol-mediated liver injury.

Paradoxically, according to other studies, HIF1-α prevents ALD. Nishiyama et al. found that ethanol-fed HIF1-α liver-specific KO mice developed more severe hypertriglyceridaemia and presented with a higher level of SREBP-1c than wild-type mice [246]. HIF1-α also protects the gut against ethanol-induced damage. Ethanol significantly inhibits intestinal HIF1-α and occludin activity, which results in thinner villi and an ecological imbalance within the intestine [247]. Intestinal epithelium-specific HIF1-α-KO mice exhibited higher serum ALT and LPS and liver TG levels and presented with more severe liver injury and increased intestinal dysbiosis after being fed an ethanol diet [248].

#### FXR

FXR, an important metabolic regulator, plays a critical role in bile acid and cholesterol metabolism and homeostasis [249, 250]. The liver-gut axis is closely involved in bile acid and cholesterol homeostasis regulation in the liver. FXR has been extensively studied as a potent therapeutic target for nonalcoholic fatty liver disease [249]. FXR regulates bile acid synthesis through two mechanisms: (i) In the liver, activation of FXR induces the expression of a small heterodimer partner (SHP) to inhibit the expression of rate-limiting enzymes in the classic pathway of bile acid synthesis, CYP7A1 and sterol 12α-hydroxylase (CYP8B1), which are involved in cholic acid synthesis, thereby inhibiting bile acid synthesis, and (ii) in the intestine, activation of FXR induces intestinal fibroblast growth factor 19 (FGF19 or mouse FGF15), which is transported to the liver via the portal vein to activate the membrane FGF receptor 4 (FGFR4)/β-Klotho complex, which activates ERK1/2 signalling in the MAPK pathway to inhibit CYP7A1 gene transcription [251–253].

Chronic ethanol exposure inactivates FXR by increasing the acetylation of FXR and interrupting the interaction between FXR and RXRα [254]. Ethanol treatment significantly alters bile acid homeostasis and the composition of the BA pool in FXR-KO mice [255]. Primary and secondary BA levels were significantly higher in FXR-KO mice, and this increase was exacerbated after ethanol treatment. The reduction in FXR activity stimulated bile acid synthesis, resulting in cholestasis [250]. Ethanol modulates bile acid synthesis in the liver through the FXR–FGF15 axis [256]. Ethanol suppresses the activity of intestinal FXR via acetylation, which decreases the production and secretion of FGF15 as well as the FGF15-driven inhibition of CYP7A1. Therefore, a regulatory cascade is established and results in the excessive production and subsequent accumulation of bile acids in the liver.

Treatment with the gut-limiting FXR agonist fexaramine reverses ethanol-induced steatosis in mouse liver [256]. Specifically, fexaramine stabilizes the intestinal barrier and protects mice against ethanol-induced liver injury [257]. This outcome suggests that ethanol-mediated downregulation of the FXR–FGF15/19 signalling axis potentially promotes the pathogenesis of ALD. A study in mice showed that long-term alcohol intake increased the bacterial choloylglycine hydrolase and unconjugated bile acid levels, decreased the Fgf15/FGF19 level, increased Cyp7a1 expression, and exacerbated AH [256]. In contrast, a recent study reported that ethanol reduced bile acid synthesis gene expression but increased the bile acid pool size by increasing the levels of intestinally reabsorbed bile acids in wild-type mice. In Cyp7a1-deficient mice, ethanol exacerbated ALD, while in Cyp7a1 transgenic mice, ethanol aggravated liver injury and inflammation [258].

Ethanol differentially regulates bile acid synthesis in humans and mice. Patients with AH present with high levels of total and conjugated bile acids and FGF19 in serum but decreased CYP7A1 and serum C4 levels, indicating suppressed bile acid synthesis [259]. In patients with alcoholic cirrhosis, serum metabolomic profiling revealed an increase in the levels of taurine and glycine-conjugated bile acids. The serum C4 level was increased, but the CYP7A1 and CYP8B1 protein expression levels were decreased [256]. Another study showed that serum G/TCDCA and G/TCA were positively correlated with ALD progression to end-state liver disease [260].

#### AhR

Aryl hydrocarbon receptor (AhR) is an aromatic hydrocarbon receptor domain-containing transcription factor that is highly expressed in the intestine, liver, and kidney. AhR can upregulate the expression of antioxidant genes in the liver. Dong et al. reported that activation of the AhR–NAD(P)H quinone dehydrogenase 1 signalling pathway increased the antioxidant capacity and led to efficient resistance to ALD [261].

By analysing the intestinal tissues of AH patients, Qian et al. observed that ethanol significantly represses the expression of AhR in intestinal epithelial cells [262]. Intestinal epithelial cell-specific KO mice were more susceptible to ethanol-induced liver injury, fat accumulation, and inflammation. In contrast, the activation of AhR exerted a protective effect on the intestinal mucosal barrier.

Through an in vitro study, Postal et al. found that the AhR agonist β-naphthoflavone stimulated the expression of intestinal TJ proteins, including occludin, tricellulin, and ZO-1 [263]. Loss of intestinal epithelial AhR enabled *Helicobacter hepaticus* and *H. ganmani* to metastasize to the mesenteric lymph nodes and liver due to damage to the intestinal barrier [262]. Therefore, ethanol may induce intestinal bacterial migration to the liver, resulting in damage through the downregulation of AhR expression. In summary, AhR activation enhances the resistance of the intestinal barrier to ethanol-induced liver injury.

AhR also regulates the expression of enzymes involved in ethanol metabolism. For instance, Munzel et al. reported that AhR promoted the expression of UGT1A6 [264]. UGT1A6 is a nonoxidative enzyme that metabolizes ethanol. This finding indicates that downregulated UGT1A6 expression due to AhR deficiency may limit the biotransformation of ethanol to water-soluble and nontoxic metabolites.

### miRNAs

MiRNAs constitute a group of small noncoding RNAs that are approximately 20 to 22 bp in length that function as important epigenetic regulators. Ethanol has been reported to trigger a variety of epigenetic events by regulating the expression of multiple miRNAs [265]. The upregulation of the miRNA (miR)-21, miR-34a, miR-155, and miR-320 and the downregulation of miR-122, miR-181a, miR-199a, and miR-200a were detected after ethanol-induced liver injury. Among these miRNAs, miR-155 and miR-122 have been the most extensively studied and thus were selected to be reviewed here.

#### miR-155

MiR-155 is an important posttranscriptional regulator and has been reported to induce steatosis and inflammation in alcohol-induced liver injury [266, 267]. Clinical studies have shown that the miR-155 level was significantly increased in the blood of ALD patients and in an ethanol-induced AH rat model [268]. In ethanol-induced liver injury, miR-155 activates the MAPK suppressor of cytokine signalling 1 (SOCS1) pathway, suppressing the signal transducer and activator of transcription 3 (STAT3) activation of proliferation-related genes, resulting in the cell cycle arrest and apoptosis of hepatocytes [269]. Notably, increasing the expression of hepatic SOCS1 mitigated the damaging effect of ethanol on the liver [270, 271].

A previous study indicated that the expression of miR-155 is also closely associated with TNF-α [272]. LPS-induced miR-155 participates in the activation of the immunoinflammatory response by mediating Kupffer cell activation and TNF-α secretion [273]. An in vitro study demonstrated that ethanol-induced miR-155 activity in RAW264.7 macrophages and Kupffer cells [274]. Ethanol may upregulate miR-155 activity by triggering the rapid degradation of IκB and releasing activated NF-κB [274]. These findings showed that, as expected, inhibition of NF-κB activation reduces ethanol-induced miR-155 activity in macrophages.

#### miR-122

MiR-122 is a liver-specific miRNA that is captured by exosomes, which are secreted from host cells. It plays a role in regulating hepatocyte differentiation, accounting for approximately 70% of liver-specific miRNAs [275]. Studies have shown that miR-122 is necessary for the maintenance of liver homeostasis [276]. Knocking out the miR-122 gene or drug inhibition of miR-122 led to an imbalance in liver lipid metabolism, iron homeostasis, and cell differentiation.

Long-term ethanol consumption exerts a direct inhibitory effect on the transcriptional regulation of miR-122 [267]. Notably, the expression level of miR-122 in the liver was significantly reduced and the degree of fat accumulation and inflammation were higher in the liver of ALD mice fed a Lieber-DeCarli liquid diet [277]. In addition, ethanol caused more severe liver damage in mice pretreated with anti-miR-122. Further evidence from this study suggested that miR-122 protected the liver against ALD probably by inhibiting the expression of HIF1-α.

## Therapeutic targets in ALD

This review highlights that multiple mechanistic systems are involved in ALD pathogenesis. Therefore, we conclude this review by proposing possible therapeutic interventions.

### Regulatory drugs targeting inflammatory factors

Oxidative stress or lipid peroxidation induced by ethanol can trigger the production of cytokines and chemokines, leading to inflammation and apoptosis. Therefore, inhibiting the inflammatory response by blocking the release of cytokines and chemokines or sequestering them is considered a promising therapeutic strategy for the treatment of ALD.

Physiological doses of IL-1β induce steatosis and increase inflammatory responses in hepatocytes. IL-1β also induces the release of the adipochemical proto-factor MCP-1 and enhances the TLR4-dependent upregulation of inflammatory signalling in macrophages [278]. Canakinumab, an IL-1β receptor agonist, and cenicriviroc, a chemokine chemoattractant cell receptor-2/5 antagonist, have been successfully developed for clinical use and may be effective for treating ALS [270, 279, 280]. Another anti-inflammatory approach involves promoting the expression of anti-inflammatory factors.

IL-22, a member of the anti-inflammatory factor family, protects the liver against oxidative stress and fibrosis, in addition to stimulating cell regeneration [281]. A previous study confirmed the therapeutic effect of the recombinant IL-22 protein drug F-652 on AH [279]. Activated M2 macrophages suppressed ethanol-induced liver inflammation and promoted tissue repair [115]. Insulin also ameliorated inflammation in ALD by inhibiting short-chain FA-induced M1 and activating M2 macrophage polarization [282].

Finally, NF-κB plays an important role in inducing the expression of inflammation-related genes. Inhibition of NF-κB by drugs such as kahweol, acanthoid acid, clomethiazole, and fucoxanthin can effectively alleviate an ethanol-induced inflammatory response and liver injury [283].

### Regulation of the balance between oxidants and antioxidants

The large amounts of ROS that are produced as a result of ethanol metabolism are the main causes of oxidative stress and lipid peroxide accumulation in the liver. Therefore, regulating the antioxidant level in the liver is a potent direction for ALD treatment. For example, exogenous supplementation with GSH or its precursors, N-acetylcysteine and S-adenosyl methionine, increased the GSH level and antioxidant capacity in hepatocytes, thus increasing liver regeneration rate [284, 285]. Additionally, lipoxygenases are critical for the oxidation of PUFAs, and lipoxygenase inhibitors may be potent therapies for chronic liver diseases due to their mediation of ethanol-induced lipid peroxidation [162].

Moreover, the pharmaceutical activation of NRF2 is thought to be a promising strategy to protect the liver from damage caused by various chemicals [286]. Protandim, an herbal dietary supplement, induces the expression of the endogenous antioxidant enzymes SOD and catalase by activating NRF2 [287, 288]. A newly synthesized drug, bardoxolone methyl, also attenuates chronic kidney diseases via its activation of NRF2 [289]. Collectively, these findings indicate that the induction of an NRF2-regulated antioxidant system is a clear option for the effective prevention or treatment of ALD.

### Targeting hepatocyte proliferation and apoptosis

Modulation of ethanol-induced cell apoptosis is considered a novel therapeutic strategy for ALD. Ethanol-induced apoptosis of liver cells can be reduced by inhibiting p53 and other apoptosis-related signalling pathway activation through drug treatment [290, 291]. Additionally, because miR-155 and miR-122 play significant roles in mediating ethanol-induced hepatocyte apoptosis, gene therapy that restores miR-122 expression may be a therapeutic alternative. For example, in mice, gene therapy that induced miR-122 activity sufficiently ameliorated ethanol-induced liver injury [277]. Finally, the administration of betaine effectively reduced homocysteine and inhibited apoptosis caused by ER stress and bile acid deposition in ALD patients [292].

### Regulation of the gut–liver axis

Ethanol can change the composition of the intestinal microbiota. Therefore, regulating the microbiota through probiotics and faecal bacteria transplantation may be potent treatments for ALD. Kirpich et al. used *Bifidobacterium bifidum* and *Lactobacillus plantarum* 8PA3 to treat AH patients [293]. Furthermore, faecal microbiota transplantation (FMT) for patients with steroid-resistant AH reduced the *Proteobacteria* abundance but increased *Firmicutes* abundance in the intestine [294]. The clinical intervention significantly increases the survival rate of patients. Therefore, increasing the use of probiotics or modifying the microbial composition in the intestine may be other therapeutic options for ALD. Clinical studies have shown that FMT is a safe approach to increasing short- and medium-term survival as well as the clinical severity scores of patients with acute chronic liver failure and severe AH.

Compounds that modulate bile acid concentrations are also possible targets. For example, ursodeoxycholic acid, a hydrophilic bile acid, can be used to protect the liver and bile duct system against bile acid toxicity by stimulating the hepatobiliary secretion of bile acids, thus prolonging cell survival [253].

## Conclusions and perspectives

The pathological process of ALD is very complex and involves a variety of potential mechanisms. While intensive studies have been devoted to understanding the pathogenesis of ALD and possible therapeutic ALD treatments, the molecular mechanisms of ALD are still incompletely understood, resulting in a limitation to the effectiveness of ALD treatment. To date, the clinical treatments of ALD have been focused on drug-promoted abstinence and behavioural interventions that reduce alcohol intake. Glucocorticoid therapy for patients with severe AH and severe ALD can prolong the survival rate [295]. However, approximately 40% of patients resistant to corticosteroids face a desperate situation for which no drug treatment is available. In addition, experimentations with antitumour necrosis factor and antioxidant therapy have led to unsatisfactory therapeutic effects [296]. Liver transplantation is the only option for patients with end-stage alcoholic liver disease, and although this intervention can prolong survival, it cannot prevent potential alcoholism and dependence, leading to the possibility of ALD recurrence [297]. Developing effective therapy for ALD has become an urgent need.

In this review, we summarize the pathogenesis of ALD and associated regulatory factors. Ethanol and its metabolic intermediate acetaldehyde are the key components causing various liver diseases. Many signalling pathways involving transcription factors, biochemical enzymes, miRNAs, and other regulatory factors can be dysregulated by ethanol and acetaldehyde, resulting in responses to oxidation, nitration, and ER stress and an imbalance between lipid metabolism and cell proliferation. Therefore, exploring possible therapeutic strategies based on the aforementioned regulators to restore the balance disrupted by alcohol is a reasonable approach. Importantly, increasing ethanol detoxification-related metabolism is likely one of the most promising strategies for preventing or curing ALD. The systematic optimization of the pathways involved in alcohol metabolism may effectively eliminate alcohol and its toxic effects.

Although alcohol can cause many malignant events in the liver, some studies have reported that appropriate alcohol intake confers a certain degree of benefit for cardiovascular protection and drug-induced liver injury. While a definitive boundary between the benign and malignant effects of alcohol remains unclear, a dose range is approximately defined by an epidemiological analysis [298]. For example, prospective cohort studies in which the dose of consumed alcohol was associated with the incidence and mortality of coronary heart disease and stroke have been performed, and the authors recommended that a dose of 2.5 to 14.9 g of alcohol per day confers modest protection against both of the analysed cardiovascular diseases, but the risk increased substantially with heavier drinking. Moreover, another study proposes that low-dose acute alcohol (0.5 g/kg) therapy has a potential protective effect on acetaminophen (APAP) drug-induced liver injury [299]. However, the influence of higher doses (over 2 g/kg) is still unclear. Consequently, we believe that the effects caused by alcohol may be dose-dependent, but that the safe dose of alcohol intake is complex and varies by geography, age, sex, and time [300]. There are still no consistent safe doses that have been reported and therefore more relevant studies are still needed.

Moreover, alcohol has been established to arouse the nervous system, has often been associated with aggressive behaviour and violence, and is a significant contributor to abnormal mortality. A study based on data published between 2013 and 2017 showed the blood concentration threshold for alcohol-induced aggression [301]. The effect of alcohol on motor impulsivity was shown to be dose-dependent, with a low dose (0.4 g/kg) of alcohol not affecting inhibitory control but a high dose of alcohol (0.8 g/kg1.15 g/L BAC) causing a reduction in inhibitory control. Additionally, low levels of alcohol were associated with impaired reflex impulses, which may lead to a higher probability of high-risk behaviour. Thus, even low-dose alcohol consumption may lead to serious harmful effects on public health and social safety in certain populations.

In summary, we believe that gaining key insights into ALD mechanisms of pathogenesis and potential therapeutic approaches relies on fully understanding the interdependence of various regulatory factors during ALD progression. The identification of central nodal regulators may be the right approach to developing an effective treatment for ALD.

## Data Availability

Not applicable.

## Abbreviations

3-NT: 3-nitrotyrosine
4-HNE: 4-hydroxynonenal
ACC: Acetyl-CoA carboxylase
ACOX-1: Acyl-CoA oxidase 1
ADH: Alcohol dehydrogenase
AFL: Alcoholic fatty liver
AH: Alcoholic hepatitis
Apo: Apolipoprotein
AhR: Aryl hydrocarbon receptor
ALD: Alcoholic liver disease
ALDH: Aldehyde dehydrogenase
ALT: Alanine aminotransferase
AMP: Adenosine 5ʹ-monophosphate
AMPK: Adenosine 5ʹ-monophosphate activated protein kinase
ASH: Alcoholic steatohepatitis
ASS: Argininosuccinate synthetase
AST: Aspartate aminotransferase
ATF: Activating transcription factor
Bcl-2: B-cell lymphoma-2
Bim: Bcl-2 interacting mediator of cell death
CAT: Catalase
CHOP: C/EBP-homologous protein
ChREBP: Carbohydrate response element binding protein
CoA: Coenzyme A
COX: Cyclooxygenase
CPT-1: Carnitine palmityl transferase-1
CREB: Camp-response element-binding protein
CCL2: C–C motif chemokine ligand 2
CYP2E1: Cytochrome P450 protein 2E1
CYP7A1: Cholesterol 7alpha-hydroxylase
eIF2α: Eukaryotic initiation factor 2α
eNOS: Endothelial nitric oxide synthase
ERK: Extracellular regulated protein kinases
ER: Endoplasmic reticulum
EtG: Ethyl glucuronide
EtS: Ethyl sulfate
FAEEs: Fatty acid ethyl esters
FAEES: Fatty acid ethyl ester synthase
FA: Fatty acid
FAS: Fatty acid synthase
FAT: Fatty acid translocase
FATP: Fatty acid transport protein
FMT: Fecal microbiota transplantation
FOXO: Forkhead box O
FXR: Farnesoid X receptor
GSH: Glutathione
GSK-3β: Glycogen synthase kinase-3β
GST: Glutathione S-transferase
HDL: High-density lipoprotein
HIF1: Hypoxia inducible factor-1
IL: Interleukin
IL-1R: IL-1 receptor
iNOS: Inducible nitric oxide synthase
IRAK: Interleukin-1 receptor-associated kinase
IRE1: Inositol-requiring enzyme 1
IκB: Inhibitory κB
JNK: C-Jun N-terminal kinase
KEAP1: Kelch-like ECH-associated protein 1
LDL: Low-density lipoprotein
LPL: Lipoprotein lipase
LPS: Lipopolysaccharide
MCP-1: Monocyte chemoattractant protein-1
MDA: Malondialdehyde
MTP: Microsomal triglyceride transfer protein
miR: micro-RNA
mTOR: Mammalian target of rapamycin
MyD88: Myeloid differentiation primary response 88
MAPK: Mitogen-activated protein kinase
NAD: Nicotinamide adenine dinucleotide
NF-κB: Nuclear factor-kappaB
NO: Nitric oxide
NRF2: Nuclear factor E2-related factor 2
NOS: Nitricoxide synthase
PC: Phosphatidylcholine
PERK: Protein kinase R (PKR)-like endoplasmic reticulum kinase
PEth: Phosphatidylethanol
PGC-1α: Peroxlsome proliferator-activated receptor-γ coactivator 1α
PI3K: Phosphatidylinositol 3-kinase
PPARα: Perixisome proliferation-activated receptor α
PUFA: Polyunsaturated fatty acids
PUMA: p53-upregulated modulator of apoptosis
ROS: Reactive oxygen species
RXR: Retinoic acid receptor
SCD1: Stearoyl-CoA desaturase 1
SHP: Small heterodimer partner
SIRT1: Sirtuin 1
SOCS1: Suppressor cytokine signaling 1c
SOD: Superoxide dismutase
SREBP: Sterol-regulatory element binding protein
SULT: Sulfotransferase
TG: Triglyceride
TJ: Tight junction
TLR: Toll-like rceptor
TNF-α: Tumor necrosis factor-α
UGT: Uridine diphospho (UDP)-glucuronosyhransferase
VLDL: Very-low-density lipoprotein
ZO-1: Zonula occludens1
